# *Chryseobacterium indologenes* Keratitis: Successful Treatment of Multidrug-Resistant Strain

**DOI:** 10.1155/2021/5527775

**Published:** 2021-05-11

**Authors:** Ivan J. Lee, Thomas Mauger

**Affiliations:** Department of Ophthalmology, West Virginia University School of Medicine, WVU Eye Institute, 1 Medical Center Dr., Morgantown, WV 26506, USA

## Abstract

A 72-year-old male with history of monocular vision with complete vision loss in his right eye from previous retinal detachment presented with 20/200 vision in the left eye with a corneal ulcer. Culture was obtained, and the patient was started on fortified tobramycin, fortified vancomycin, and amphotericin. Despite the antibiotics, the patient did not significantly improve, after which another culture was obtained before the patient was taken to the surgery for cryotherapy and a partial conjunctival flap. The culture identified *Chryseobacterium indologenes*. There have been fewer than a handful of cases reported in the last three decades with different antibiotic susceptibility profiles. Our patient was successfully treated with ciprofloxacin and ceftazidime with the final vision of 20/40.

## 1. Introduction

*Chryseobacterium indologenes* is an aerobic, gram-negative bacillus that is ubiquitous in nature but is rarely present in the human microflora [1]. While *C. indologenes* is a rare pathogen known to cause different types of infections including bacteremia, meningitis, pneumonia, and indwelling device-associated infections [2], only a few cases of keratitis have been reported to date [3–5], each with varying antibiotic susceptibility profile and clinical course. In this case, we present *C. indologenes* keratitis successfully treated with surgical and pharmacological interventions.

## 2. Case

**Figure 1:**
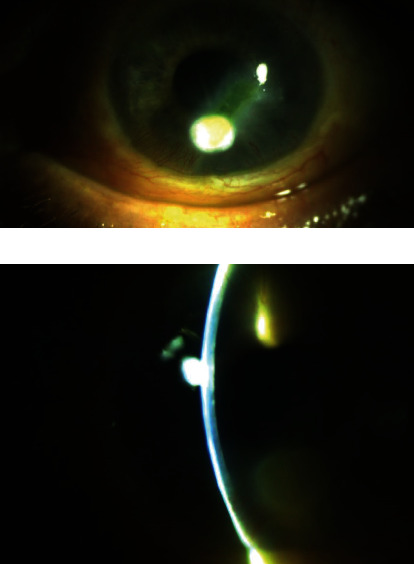
Bacterial keratitis at presentation.

**Figure 2:**
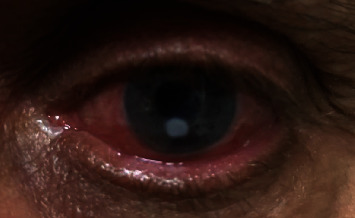
Patient at follow-up with 20/40 vision with persisting but improving infiltrate. Partial conjunctival flap is rotated inferiorly.

A 72-year-old male with history of chronic adrenal insufficiency, diabetes mellitus, and monocular vision with complete vision loss in his right eye from previous retinal detachment presented with burning eye pain, redness, and decreased vision of his left eye over a 3-week period. On the initial exam, the patient had 20/200 vision with external findings significant for 3 mm dense round infiltrate in the inferocentral cornea with overlying epithelial defect extending superotemporally. Anterior chamber reaction or hypopyon was not observed (Figure 1). The patient was admitted to the hospital and was empirically started on hourly fortified tobramycin 15 mg/ml, fortified vancomycin 50 mg/ml, and amphotericin 0.15% after cultures were performed. Despite the initial treatment for five days, the patient did not significantly improve with no growth in the initial cultures. The decision was made to obtain another set of cultures after holding antibiotic drops for one day, and the patient was started on tobramycin, vancomycin, moxifloxacin, and natamycin eye drops. While awaiting on the second set of cultures, the patient was taken to the surgery for cryotherapy and a partial conjunctival flap due to minimal clinical improvement despite the aforementioned medical management. The patient did well postoperatively with improvement in his symptoms. During this time, the cultures identified *Chryseobacterium indologenes* as the causative organism. A chocolate agar plate was used to isolate the species. A matrix-assisted laser desorption/ionization-time-of-flight mass spectrometer (MALDI-TOF) was implemented for the identification of the species with 99.9% match. The Kirby-Bauer susceptibility test protocol via a disk diffusion method was applied, in which zone size was measured and interpreted according to the Clinical and Laboratory Standards Institute (CLSI) Performance Standards for Antimicrobial Susceptibility Testing 31^st^ edition (Table 1). The medications were switched to topical ciprofloxacin and ceftazidime accordingly. The flap has rotated fully about 2 weeks after the surgery, exposing 1.7 mm infiltrate with 3 mm epithelial defect overlying the part of the infiltrate. Over the next month, the patient showed slow clinical improvement. An external exam showed gradual clearing of the infiltrate as well as reepithelialization of the surface. At this point, prednisolone acetate 1% was added to his medication regimen. The patient's vision ultimately improved to 20/40, two months after the initial presentation (Figure 2).

## 3. Discussion

*Chryseobacterium indologenes*, formerly known as *Flavobacterium indologenes* or *Flavobacterium aureum*, is an aerobic, nonfermentative, oxidase-positive, and indole-positive gram-negative bacillus that is widely distributed in nature, although rarely present in the human microflora [1]. *C. indologenes* is a rare pathogen known to cause different types of infections including bacteremia, meningitis, pneumonia, and indwelling device-associated infections [2]. Risk factors associated with *C. indologenes* infection includes older age, immunocompromised clinical status including diabetes or systemic steroid treatment, and history of indwelling catheter [6–10], which correlate with our patient with diabetes and chronic steroid treatment for adrenal insufficiency.

The pathogenicity of *Chryseobacterium* species is postulated to involve endotoxin [11, 12] and biofilms [13]. Endotoxins as well as elastase enzymes released by the microorganism cause collagen breakdown, cascading into inflammatory responses of the cornea and eventually corneal perforation if left untreated. The biofilm allows the formation of microbial community that may attach to the solid surface surrounded by extracellular polymeric substances produced by the microorganisms, especially in the setting of indwelling catheter, corroborating the relationship between its mode of virulence and predisposing factor to infection [13]. In addition, *C. indologenes* is intrinsically resistant to carbapenems and cephalosporins by producing molecular class A *β*-lactamase and class B carbapenem-hydrolyzing *β*-lactamase (IND1-IND7) [14–17], providing multidrug resistance by nature.

*C. indologenes* have been involved in the development of keratitis. A 47-year-old Asian male suffered corneal perforation despite fortified topical gentamicin and cefazolin for presumed *Pseudomonas aeruginosa* infection. The culture later isolated *C. indologenes* with multiple drug resistance except intermediate response to ceftazidime, which ultimately resolved with hourly ceftazidime eye drops for three weeks [3]. Another 83-year-old female was treated with fortified vancomycin and ceftazidime for bacterial keratitis whose culture ultimately grew *C. indologenes* which was highly resistant to all antibiotics except for trimethoprim-sulfamethoxazole with clinical improvement after one month of the antibiotics [4]. For both cases, susceptibility of strains to antibiotics differed from the strain isolated from our patient, which suggests the standardized treatment for *C. indologenes* to be challenging.

In conclusion, *Chryseobacterium indologenes* is a possibly emerging bacterial cause of keratitis that should be considered for recalcitrant cases in older, immunocompromised groups of patients. Standardization of antimicrobial treatment for *C. indologenes* keratitis remains difficult due to varying susceptibility to antibiotics on a few number of cases reported.

## Figures and Tables

**Table 1:** Susceptibility profile for *Chryseobacterium indologenes*.

Antibiotic	Susceptibility	Disc size for Kirby-Bauer susceptibility^∗^
Amikacin	Resistant	13 mm
Aztreonam	Resistant	6 mm
Cefepime	Sensitive	24 mm
Ceftazidime	Intermediate	17 mm
Ciprofloxacin	Sensitive	28 mm
Gentamicin	Resistant	12 mm
Meropenem	Sensitive	26 mm
Piperacillin/tazobactam	Sensitive	31 mm
Tobramycin	Resistant	6 mm

^∗^Note that the disc size for determination of susceptibility varies for each species.

## Data Availability

Data are available on request.
